# Impact of Selenium and Vitamin E Deficiency on Zika Virus Pathogenesis and Immune Response in Mice

**DOI:** 10.3390/v18020177

**Published:** 2026-01-28

**Authors:** Olukunle O. Oluwasemowo, Monica E. Graham, James B. Thissen, Aram Avila-Herrera, Jeffrey A. Kimbrel, Deepa K. Murugesh, Dina R. Weilhammer, Tanya Tanner, Nicole M. Collette, Monica K. Borucki

**Affiliations:** 1 Biosciences and Biotechnology Division, Physical and Life Sciences Directorate, Lawrence Livermore National Laboratory, P.O. Box 808, Livermore, CA 94550, USA; oluwasemowo1@llnl.gov (O.O.O.); graham71@llnl.gov (M.E.G.); collette2@llnl.gov (N.M.C.); 2 Global Security Computing Applications Division, Computing Directorate, Lawrence Livermore National Laboratory, P.O. Box 808, Livermore, CA 94550, USA

**Keywords:** Zika virus, selenium, vitamin E, mutation, nutrition, cytokine, neuroinvasion, quasispecies

## Abstract

Micronutrient status is recognized to influence host susceptibility to viral infections, yet its impact on Zika virus (ZIKV) pathogenesis remains incompletely understood. We investigated the effects of dietary selenium and combined selenium plus vitamin E deficiency on ZIKV infection outcomes in a type I interferon α/β receptor knockout (Ifnar1^−/−^) murine model. Mice maintained on deficient diets exhibited significantly lower neutralizing antibody titers and reduced levels of key antiviral cytokines (IFN-γ, TNF-α, IFN-α, IFN-β, IL-12p70, CCL5) compared to controls. Correspondingly, higher viral RNA loads were detected in the brains of double-deficient mice, which also experienced greater weight loss and increased mortality. Deep sequencing revealed no major differences in overall viral genome diversity across diet groups; however, specific mutations, including V330L and D67E in the E gene, and V360I in the NS3 gene, were enriched or detected in nutritionally deficient animals. These findings suggest that antioxidant micronutrient deficiency impairs both humoral and cellular immune responses to ZIKV, potentially facilitating enhanced neuroinvasion. While the functional consequences of the identified mutations warrant further investigation, our results underscore the importance of adequate micronutrient intake for optimal antiviral defense. Further studies are needed to clarify the epidemiological significance of these observations.

## 1. Introduction

Selenium and vitamin E are essential micronutrients that function synergistically as critical components of the host antioxidant defense system [1,2,3,4]. The impact of selenium and/or vitamin E deficiency on viral pathogenesis has been extensively documented through both experimental and clinical studies, thereby demonstrating that nutritional deficiencies can fundamentally alter host–pathogen interactions and disease outcomes [5,6,7,8,9,10,11,12]. Geographic regions characterized by selenium-depleted soils have consistently reported more severe manifestations of infectious diseases, including viral infections [13,14], as well as more pronounced noninfectious disease outcomes [15,16]. In contrast, targeted supplementation with selenium and/or vitamin E has shown therapeutic benefits by improving disease progression and clinical outcomes [17,18,19].

Selenium is an essential cofactor for more than 25 selenoproteins, which are involved in a wide range of biological processes, including redox signaling, antioxidant defense, thyroid hormone metabolism, and regulation of both innate and adaptive immune responses [20,21]. Among these, glutathione peroxidases, thioredoxin reductases, and selenoprotein P are particularly critical for maintaining cellular redox homeostasis and protecting tissues from oxidative stress [22]. Vitamin E, primarily in the form of α-tocopherol, acts as a potent lipid-soluble antioxidant, neutralizing reactive oxygen species and preserving membrane integrity [23]. The interdependent relationship between selenium and vitamin E is exemplified by their ability to compensate for each other’s antioxidant activities, with selenium-dependent glutathione peroxidases (GPx) protecting against lipid peroxidation when vitamin E levels are inadequate [24,25] and vitamin E partially compensating for the loss of selenium-dependent GPx4 [1,26].

Emerging evidence suggests that climate change is exacerbating global selenium deficiency by altering precipitation patterns and soil chemistry, thereby reducing selenium bioavailability in many regions [27,28]. This environmental shift has far-reaching consequences for human and animal health, as selenium deficiency not only impairs antiviral immune responses but also accelerates the emergence of more virulent viral variants by increasing viral mutation rates and immune selection pressure [29]. This phenomenon is particularly pronounced in RNA viruses, which inherently possess high mutation rates due to the error-prone nature of their RNA-dependent RNA polymerases, leading to the rapid generation of genetically diverse viral populations or quasispecies [30,31]. Under conditions of oxidative stress associated with micronutrient deficiencies, RNA virus mutation rates can increase dramatically, creating evolutionary bottlenecks that select for variants with enhanced virulence, immune evasion capabilities, or altered tissue tropism [32]. These findings underscore a critical intersection between environmental change, nutritional status, and infectious disease dynamics. Predictive modeling indicates that climate-driven selenium deficiency may continue to expand globally, potentially affecting billions and creating ecological conditions favorable for viral evolution thereby increasing the risk of future pandemics [27,33].

Zika virus (ZIKV), a mosquito-borne flavivirus closely related to yellow fever virus, West Nile virus, and dengue virus, has emerged as a significant global health threat, especially following the devastating 2015–2016 pandemic that affected over 70 countries and territories [34,35]. As a positive-sense single-stranded RNA virus, ZIKV inherently exhibits a high mutation rate due to the lack of proofreading activity in its RNA-dependent RNA polymerase, resulting in the formation of genetically diverse viral quasispecies within infected hosts [36,37]. This genetic plasticity enables rapid adaptation to selective pressures, including those imposed by host immunity and antiviral treatments [38,39].

Clinically, ZIKV infection is associated with severe neurological complications, including microcephaly in developing fetuses, Guillain-Barré syndrome in adults, and other neurotropic manifestations [40,41]. The virus’s capacity for rapid geographic spread, neurotropism, persistence in immune-privileged sites, and propensity for genetic evolution has led the World Health Organization to designate ZIKV as a priority pathogen with significant re-emergence and pandemic potential [42,43]. Importantly, the evolutionary dynamics of RNA viruses like ZIKV are highly sensitive to host nutritional status, as oxidative stress associated with micronutrient deficiencies can further increase mutation rates and facilitate the selection of variants with enhanced virulence or immune escape [29,44].

Despite extensive research on ZIKV pathogenesis, the molecular mechanisms by which micronutrient deficiencies influence viral evolution, tissue tropism, and host immune responses remain poorly understood. The combination of intrinsic genetic instability in RNA viruses and the oxidative stress environment created by selenium and vitamin E deficiency may accelerate viral mutation rates and promote the emergence of variants with altered tissue tropism, enhanced virulence, or increased pandemic potential [32,36]. Given the established role of these micronutrients in antiviral immunity and the global trend toward increasing deficiency states, investigating their impact on ZIKV evolution and pathogenesis addresses a critical knowledge gap with significant implications for pandemic preparedness and therapeutic intervention strategies.

This study aimed to elucidate the genotypic, immunological, and phenotypic changes associated with ZIKV infection in hosts experiencing combined selenium and vitamin E deficiency, with particular emphasis on understanding how nutritional status influences viral tissue distribution, disease severity, and the potential emergence of viral variants with enhanced pathogenic properties and pandemic potential.

## 2. Materials and Methods

### 2.1. Virus Stocks

Two Asian lineage ZIKV strains were utilized for murine infection in this study: the epidemic strain PRVABC59 (isolated in Puerto Rico, 2015) and the pre-epidemic strain FSS13025 (isolated in Cambodia, 2010). These strains were selected for their direct relevance to recent global outbreaks and to minimize potential confounding effects of inter-lineage variability on infection outcomes. The PRVABC59 strain (Human/2015/Puerto Rico) was obtained from BEI Resources (NR-50240) while the FSS13025 strain was obtained from the University of Texas Medical Branch (UTMB) Arbovirus Reference Collection. Viral stocks were prepared by one or two passages in Vero cells. Infectious titers were determined using plaque assay method, as previously described [45].

### 2.2. Mouse Diets

Three distinct dietary regimens were used for the experimental mice: a nutritionally adequate, normal diet (ND), a selenium-deficient diet (SD), and a selenium plus vitamin E double-deficient diet (SED). All diets were specially formulated and commercially supplied by TEKLAD (TD.92163). The expected selenium content of these diets is based on previously published analyses of similar formulations from the same vendor [46], which demonstrated that the Se-adequate diet aligns with selenium levels considered sufficient in humans, while the Se-deficient and SED diets correspond to levels associated with deficiency [47]. Although direct measurement of selenium concentrations in our dietary batches was not performed, we confirmed the functional adequacy of the ND by assessing higher GPx activity [48] in the sera of mice fed this diet compared to the SD and SED groups. Detailed compositions of each diet are provided in the Supplemental Information (Supplementary Table S1).

### 2.3. In Vivo Infections

ZIKV does not suppress type I interferon (Ifnar1) expression in mice as it does in humans, rendering wild-type mice resistant to disease. To overcome this limitation, we utilized a mouse strain lacking the type I interferon α/β receptor (Ifnar1^−/−^), which is susceptible to ZIKV infection and widely used for studies of ZIKV pathogenesis [49,50].

Type I interferon α/β receptor knockout (Ifnar1^−/−^) mice, approximately three weeks old, were obtained from Jackson Laboratories (Bar Harbor, ME, USA) and housed in the Animal Care Facility at Lawrence Livermore National Laboratory (LLNL) under protocols approved by the LLNL Institutional Animal Care and Use Committee (approval number 22-10-058 (315)).

Mice were randomly assigned to three dietary cohorts. The control group received a nutritionally complete (normal) diet (ND), while experimental groups were fed either a selenium-deficient diet (SD) or a selenium and vitamin E double-deficient diet (SED). Diets were administered for 5 to 6 weeks prior to infection to ensure depletion of respective micronutrient stores, as previously described [51] with the control group maintained on the normal diet for the same duration.

Micronutrient status was assessed by measuring glutathione peroxidase (GPx) activity and alpha-tocopherol (TCPa) concentrations in mouse serum, serving as proxies for selenium and vitamin E levels, respectively. GPx activity was determined using the Glutathione Peroxidase Assay Kit (MAK437-1KT; Sigma-Aldrich, St. Louis, MO, USA) according to the manufacturer’s instructions and serum TCPa concentrations were quantified using the General Alpha-Tocopherol (TCPa) ELISA Kit (MBS2000369; MyBiosource, San Diego, CA, USA) following the manufacturer’s protocol.

For infection, virus stocks containing approximately 3.8 × 10^6^ PFU/mL were used, with sterile phosphate-buffered saline (PBS) serving as the mock infection control. Mice were lightly anesthetized with isoflurane prior to subcutaneous administration of 0.05 mL of the virus stock into the right hind limb. Baseline body weights were recorded at the time of infection, and subsequent weights were measured daily until the termination of the experiment. All animals were observed at least once daily for clinical signs of illness, including lethargy, ruffled fur, hunched posture, and neurological symptoms such as paralysis and tremors. Mortality was documented, and any mouse exhibiting a weight loss exceeding 20% of its baseline value was humanely euthanized prior to the scheduled experimental endpoint.

### 2.4. Sample Collection

Sample collection was performed at two predetermined timepoints, corresponding to peak viremia 6 days post-infection, (dpi) and tissue pathology (14 dpi). At each experimental endpoint, mice were humanely euthanized using controlled atmosphere CO_2_ stunning in accordance with institutional guidelines. At 6 dpi, whole blood samples were collected via cardiac puncture for nucleic acid extraction, viral genome quantification, deep sequencing analysis and immunological cytokine profiling. At 14 dpi, blood samples were collected for neutralizing antibody quantification. In addition, brain and reproductive tissues (ovaries in females, testes in males) were harvested at 14 dpi for further analysis.

For molecular evaluation, tissue samples were preserved in RNAlater solution (Invitrogen, Carlsbad, CA, USA) for subsequent nucleic acid extraction, viral genome quantification, and deep sequencing. All sample collection procedures were performed under aseptic conditions. Samples designated for molecular analysis were either processed immediately or stored at −80 °C and processed at the earliest possible time to maintain sample integrity and minimize RNA degradation.

### 2.5. Sample Processing and RNA Extraction

Whole blood samples were allowed to clot at room temperature for 30 min and subsequently centrifuged at 1500× *g* for 10 min at 4 °C to separate serum. The serum fraction was carefully collected and stored at −80 °C until nucleic acid extraction.

Tissues preserved in RNAlater were removed and excess solution was blotted away. Approximately 30 mg of reproductive tissue (testes or ovaries) and 50 mg of brain tissue were weighed and transferred into sterile tubes containing 3 mm ceramic beads. Each sample was lysed in 600 μL of RLT buffer (Qiagen, Hilden, Germany) supplemented with β-mercaptoethanol, then homogenized using an Omni Bead Ruptor homogenizer according to the manufacturer’s protocol.

Total RNA was extracted from homogenized tissues using the RNeasy Mini Kit (Qiagen, Hilden, Germany) with on-column DNase I treatment (Qiagen) to remove genomic DNA. RNA was eluted in 65 μL of RNase-free water and stored at −80 °C until further analysis. For serum samples, RNA was extracted from 140 μL of serum using the RNeasy Mini Kit (Qiagen) according to the manufacturer’s instructions. RNA was eluted in 65 μL of RNase-free water and stored at −80 °C.

### 2.6. Quantification of Viral RNA Genome

ZIKV genome copy numbers were determined using one-step quantitative reverse transcription PCR (RT-qPCR). Reactions were performed with PrimeTime One-Step RT-qPCR Master Mix (Integrated DNA Technologies, Coralville, IA, USA) using ZIKV-specific primers and probe: forward primer ZIKV_835, reverse primer ZIKV_911c, and probe ZIKV_860 [52]. Absolute quantification was achieved by generating a standard curve from a synthetic RNA standard of known concentration (Genscript, Piscataway, NJ, USA).

RT-qPCR was performed under the following thermal cycling conditions: initial reverse transcription at 98 °C for 30 s, followed by 40 cycles of denaturation at 98 °C for 15 s, annealing at 64 °C for 20 s, and extension at 72 °C for 1.2 min, with a final extension at 72 °C for 10 min. All samples were analyzed in duplicate. Viral genome copies per microliter of serum or milligram of tissue were calculated after correcting for serum volume or tissue weight, respectively.

### 2.7. Immunological Cytokines Profiling

To evaluate the effect of dietary selenium and vitamin E deficiency on the immune response to ZIKV infection, serum cytokine levels were analyzed in mice maintained on the three diets: ND, SD, and SED. Blood samples were collected at 6 dpi from ZIKV-infected mice, while blood from mock-infected (PBS-injected) mice in each dietary group served as controls.

Serum cytokine concentrations were determined using the LEGENDplex™ Mouse Anti-Virus Response Panel 13-plex assay (BioLegend, San Diego, CA, USA), following the manufacturer’s protocol. Briefly, serum samples collected at 6 dpi were thawed on ice and diluted two-fold with Matrix A reagent provided in the kit. Flow cytometric analysis was performed using a BD FACSCelesta™ Cell Analyzer (BD Biosciences, Franklin Lakes, NJ, USA). Instrument settings and compensation were established using the kit’s setup and control beads in accordance with the LEGENDplex™ protocol. For each sample, a minimum of 300 events per analyte region was acquired to ensure statistical reliability.

Raw flow cytometry data were processed using the LEGENDplex™ Data Analysis Software Suite (https://www.biolegend.com/en-ie/immunoassays/legendplex/support/software, accessed on 12 September 2025), which enabled automated gating, standard curve generation, and quantification of cytokine concentrations. The 13-plex panel measured the following cytokines: IFN-α, IFN-β, IFN-γ, IL-6, TNF-α, IL-1β, IL-10, IL-12p70, IP-10 (CXCL10), MCP-1 (CCL2), CXCL1, CCL5, and GM-CSF. Cytokine levels from ZIKV-infected mice were normalized to those from PBS-injected controls within each diet group.

### 2.8. Plaque Reduction Neutralization Test (PRNT)

The PRNT assay was performed as previously described [53], with modifications as detailed below. Vero/TMPRSS2 cells [45] were cultured in 24-well plates for the assay. Serum samples collected from mice 14 days post ZIKV infection was used. Six samples were randomly selected from each dietary group: ND, SD, and SED. Blood samples were allowed to clot, and serum was separated by centrifugation. Vero/TMPRSS2 cells were seeded in 24-well plates and grown to 80–90% confluency. Serum samples were heat-inactivated at 56 °C for 30 min. Serial three-fold dilutions of each serum sample were prepared in DMEM. Equal volumes of diluted serum and ZIKV (at a predetermined concentration to achieve 30–50 plaques per well in the virus control) were mixed and incubated at 37 °C for 1 h to allow neutralization. After incubation, 200 µL of the virus-serum mixture was added to each well and incubated at 37 °C for 1 h with gentle rocking every 15 min. The inoculum was then removed, and cells were overlaid with DMEM containing 1% methylcellulose and 2% FBS. Plates were incubated at 37 °C in 5% CO_2_ for 4–5 days. Following incubation, cells were stained with 1% crystal violet to visualize plaques. Plaques were counted, and the percentage of plaque reduction was calculated relative to virus-only controls. The PRNT_50_ titer was defined as the highest serum dilution resulting in a 50% reduction in plaque number compared to the virus control. All samples were assayed in duplicate, and appropriate negative and positive controls were included in each run.

### 2.9. Amplicon-Based ZIKV Sequencing

#### 2.9.1. Primer Design

A custom primer set (designated M1000 primers) was designed for multiplex amplification of ZIKV strains PRVABC59 and FSS13025 using the Primal Scheme v3.3.0 web-based primer design tool (https://primalscheme.com/, accessed on 21 July 2023) [54]. The set included 12 primer pairs, each generating amplicons of approximately 1000 nucleotides with overlaps of 114–157 nucleotides, ensuring complete coverage of the ~11 kb ZIKV genome. In a pilot study, the M1000 primers demonstrated improved genome coverage compared to previously published primer sets [55] as shown in Supplementary Figure S2. Primer sequences are provided in the Supplementary Materials (Supplementary Table S2).

#### 2.9.2. RT-PCR

Whole-genome sequencing of ZIKV was performed using an amplicon-based approach with the M1000 primers, following a protocol similar to Grubaugh et al. [55]. First-strand cDNA synthesis was performed using 7 μL of extracted RNA and the LunaScript^®^ RT SuperMix Kit (New England Biolabs, Ipswich, MA, USA) with the following conditions: 25 °C for 10 min, 50 °C for 10 min, 85 °C for 5 min, then held at 4 °C.

For PCR amplification, 2.5 μL of cDNA per reaction was used. The 12 primer pairs were divided into two pools (Pool 1 and Pool 2), each containing 6 primer pairs prepared from equal volumes of 10 μM primer stocks. Two multiplex PCR reactions were set up for each sample, one for each primer pool, using 2.5 μL of the respective primer pool. PCR was performed with Q5 High-Fidelity DNA Polymerase (New England Biolabs) under the following cycling conditions: initial denaturation at 98 °C for 30 s, followed by 35 cycles of 95 °C for 15 s and 65 °C for 5 min, with a final hold at 4 °C. Amplification success was confirmed by electrophoresis of 5 μL of PCR products on 1% agarose gels.

#### 2.9.3. Library Preparation and Sequencing

Amplicons from the ZIKVprimer pools were combined in equal volumes to create a total of 20 μL pooled product per sample. Pooled amplicons were purified using 0.7× AMPure XP magnetic beads (Beckman Coulter, Brea, CA, USA) according to the manufacturer’s standard protocol and eluted in 22 μL of nuclease-free water. Purified PCR products were quantified using a Qubit 4.0 fluorometer (Thermo Fisher Scientific, Waltham, MA, USA).

Sequencing libraries were prepared using 50 ng of purified amplicons as input for the Illumina DNA Prep library preparation kit (Illumina, San Diego, CA, USA), following the manufacturer’s protocol for small amplicons. Completed libraries were quantified and quality-assessed using the TapeStation 4200 system (Agilent Technologies, Santa Clara, CA, USA). Libraries were normalized to 4 nM, pooled in equimolar ratios, and diluted to a final loading concentration of 750 pM for sequencing on the Illumina NexSeq 2000 platform using a P1 300 cycle (2 × 150 bp) kit with onboard denaturation and dilution. Raw sequencing data have been deposited in the NCBI Sequence Read Archive under BioProject accession number PRJNA1374194.

### 2.10. Bioinformatics Analysis

Variant calling and sequence analysis were performed using Mappgene (https://github.com/LLNL/mappgene, accessed on 21 July 2025), a variant calling pipeline designed for high-performance computing environments [56], using the parameters listed in Supplementary Table S3 The Mappgene pipeline integrates multiple bioinformatics tools: BWA-MEM v0.7.17 for read alignment to the ZIKV reference genome (PRVABC59, GenBank: KU501215.1), and LoFreq v2.1.5 for additional variant detection.

Raw sequencing reads underwent quality control and preprocessing, and high-quality reads were aligned to the reference genome using default BWA-MEM parameters. Post-alignment processing included automated primer sequence removal with iVar’s region-based, strand- and quality-aware trim command. Read pairs in which either mate contained a substitution or low-quality insertion in the primer region were removed using iVar ‘getmasked’ and ‘removereads’. Simple plurality consensus sequences were generated with iVar consensus, requiring a minimum depth of 10 reads per position; sites with fewer than 10 reads were assigned as N. Variant calling was performed with LoFreq, which uses an error model that incorporates base quality and sequencing depth. Variants were initially identified using permissive default filters: variant quality Phred score > 20, minimum coverage of 10 reads, and strand bias defined as FDR-adjusted *p* < 0.001 with at least 85% of reads on a single strand. The resulting calls were then filtered to retain only variants with an allele frequency ≥ 1%. 

### 2.11. Statistical Analysis

Statistical analyses were performed using GraphPad Prism version 10.0 (GraphPad Software, San Diego, CA, USA) and R version 4.5.0 [57]. Data are presented as mean ± standard deviation, unless otherwise indicated. Viral load comparisons across multiple groups were analyzed using two-way analysis of variance (ANOVA) followed by Tukey’s multiple comparison test. Glutathione peroxidase (GPx) activity was assessed using the Kruskal-Walli’s test. Longitudinal weight data were evaluated using a mixed-effects model with restricted maximum likelihood (REML) estimation. Neutralizing antibody titers (PRNT) were analyzed using ordinary one-way ANOVA with appropriate post hoc comparisons. For cytokine analyses, comparison of cytokine levels between PBS-treated and ZIKV-infected mice within each diet group was performed using two-way ANOVA with Šídák’s multiple comparisons test. Comparison of individual cytokine expression between infected mice from different diet groups was performed after log10 (logY) transformation using ordinary one-way ANOVA with Tukey’s multiple comparisons test. Statistical significance was defined as *p* < 0.05. All tests were two-tailed.

## 3. Results

A total of 120 Ifnar1^−/−^ mice were used in this study, with equal numbers allocated to two experimental endpoints: day 6 and day 14 (Table 1). For the day 6 endpoint, 60 serum samples were collected. For the day 14 endpoint, 180 samples were obtained, comprising serum, brain, and reproductive tissues from each mouse.

### 3.1. GPx Activity and Vitamin E Levels in Nutritionally Deficient Groups

**Figure 1:**
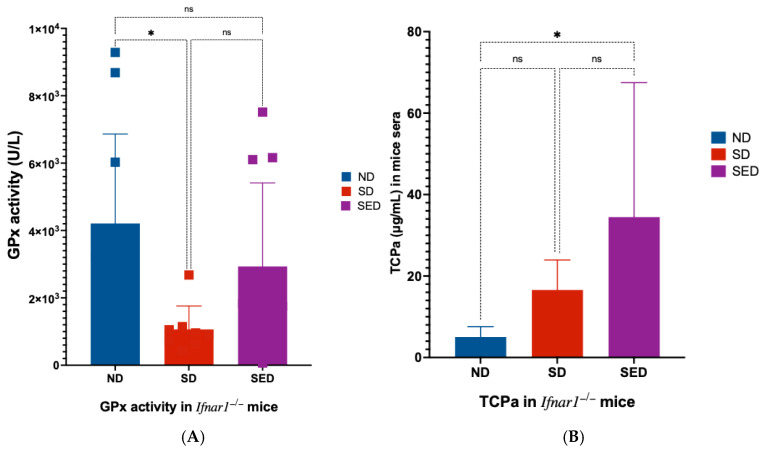
Serum selenium and vitamin E status in mice after dietary intervention. (**A**) Glutathione peroxidase (GPx) activity and (**B**) alpha-tocopherol (TCPa) concentration in the serum of mice after more than five weeks on normal diet (ND), selenium-deficient diet (SD), or selenium and vitamin E-deficient diet (SED). TCPa levels were measured by competitive ELISA, with higher assay signal indicating lower vitamin E concentration. ns: not significant; *: *p* < 0.05.

Glutathione peroxidase (GPx) activity is highly dependent on selenium levels, since selenium is required for its catalytic function [4,58]. Therefore, measurement of GPx activity provides a sensitive and specific readout of selenium status in the experimental mouse cohorts. Serum GPx activity was significantly reduced in mice fed the selenium-deficient (SD) diet compared to the normal diet (ND) group (*p* = 0.0145; Figure 1A). Mice maintained on the selenium and vitamin E-deficient (SED) diet showed similar trend of reduced GPx activity relative to the ND group. Serum alpha-tocopherol (TCPa) concentrations were assessed using a competitive ELISA, in which higher detected TCPa signals correspond to lower actual vitamin E concentrations in the sera. Accordingly, the SED group exhibited the highest TCPa signal, reflecting the lowest level of vitamin E among the groups (*p* = 0.0493; Figure 1B).

### 3.2. Severity of ZIKV Infection in Selenium and Vitamin E Double-Deficient Mice

**Figure 2:**
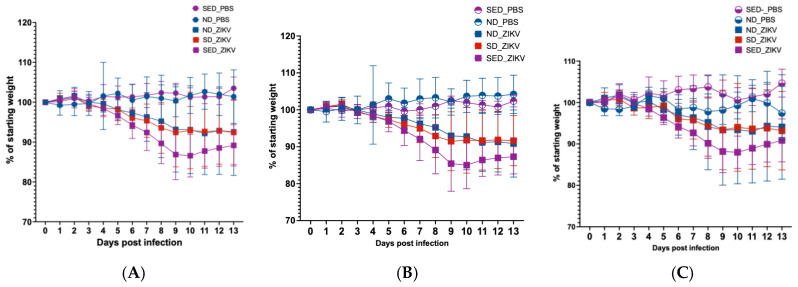
Percentage weight change in mice during ZIKV infection. Average daily weight is shown as percentage of starting weight for each group throughout the course of infection. (**A**) Combined male and female data. (**B**) Male only. (**C**) Female only. Groups are defined as follows: SED_PBS: PBS-injected mice on selenium and vitamin E double-deficient diet; ND_PBS: PBS-injected mice on normal diet; ND_ZIKV: Zika virus-infected mice on normal diet; SD_ZIKV: Zika virus-infected mice on selenium-deficient diet; SED_ZIKV: Zika virus-infected mice on selenium and vitamin E double-deficient diet.

Body weight loss is commonly used as a marker for disease severity in ZIKV-infected mice [59,60]. We hypothesized that selenium and vitamin E deficiency, will lead to a more severe ZIKV disease outcome, especially, since deficiencies in these micronutrients have been shown to exacerbate the severity of viral infections in animal models [8,61]. Body weight remained stable in all PBS-injected mice, regardless of diet (Figure 2A). In contrast, Zika virus-infected mice exhibited significant weight loss (*p* < 0.001) beginning at 3 dpi, with the greatest decline at 9–10 dpi. Zika-infected mice on the normal diet (ND_ZIKV) and selenium-deficient diet (SD_ZIKV) experienced similar weight loss (*p* = 0.1616). However, Zika-infected mice on the selenium and vitamin E double-deficient diet (SED_ZIKV) lost the most weight, and three mice in this group died at 9 dpi. Weight loss patterns in the combined male and female data (Figure 2A) were consistent when males (Figure 2B) and females (Figure 2C) were analyzed separately.

### 3.3. ZIKV RNA Levels in Brain Tissue of Selenium and Vitamin E Double-Deficient Mice

ZIKV viremia typically peaks within the first week after infection [59,60], and preliminary studies in our laboratory identified day 6 post-infection as the peak of viremia in our model. Preliminary experiments also demonstrated that the PRVABC59 strain yielded significantly higher viral genome copies in sera of mice compared to FSS13025 (Supplemental Figure S1). Consequently, subsequent experiments were conducted exclusively with the PRVABC59 strain. There is substantial evidence that ZIKV can persist in immune-privileged organs (including the brain and reproductive tissues) beyond the acute phase, contributing to neurological and reproductive pathologies [62,63,64]. We hypothesized that selenium and vitamin E deficiency could aggravate ZIKV systemic replication and persistence. Therefore, we measured ZIKV RNA in the blood at 6 dpi, and in the brain, ovaries, and testis tissues collected from mice at 14 dpi.

**Figure 3:**
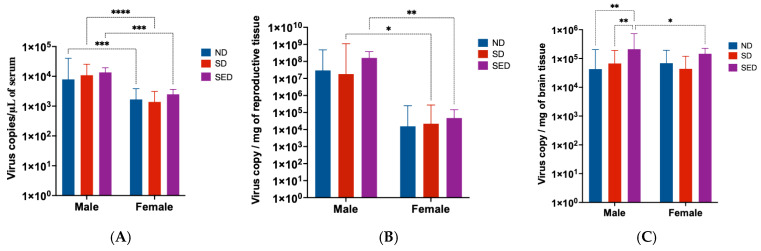
ZIKV RNA genome copies in blood, reproductive, and brain tissues of infected mice. (**A**) ZIKV genome copies in blood of mice at 6 days post infection. (**B**) ZIKV genome copies in reproductive tissues (testes and ovaries) of mice at 14 days post infection. (**C**) ZIKV genome copies in brain tissue of mice at 14 days post infection. ND: Normal diet. SD: Selenium-deficient diet. SED: Selenium and vitamin E double-deficient diet. Only significant *p* values are shown on the figure. *: *p* < 0.05; **: *p* < 0.01; ***: *p* < 0.001; ****: *p* < 0.0001.

At 6 dpi, viral RNA levels in the blood (Figure 3A) were significantly higher in male mice than in females across all diet groups (*p* ≤ 0.0001). However, within each sex, no significant differences in blood viral titers were observed between the different diet groups. In reproductive tissues (Figure 3B), viral genome copies were consistently higher in the testes compared to the ovaries, regardless of dietary group. No significant differences in viral loads were found among the diet groups for either sex. A distinct pattern emerged in brain tissues (Figure 3C). ZIKV RNA levels in the brains of mice on the selenium and vitamin E double-deficient diet (SED) were substantially higher than those in mice on the normal (ND) or selenium-deficient (SD) diets. This increase was observed in both male and female mice but was statistically significant for male mice only (*p* ≤ 0.0082). In contrast, viral genome copies in the brains of ND and SD groups were similar between sexes and remained much lower than those seen in the SED group.

### 3.4. Genome Diversity and Mutation Analysis Across Dietary Groups

**Figure 4:**
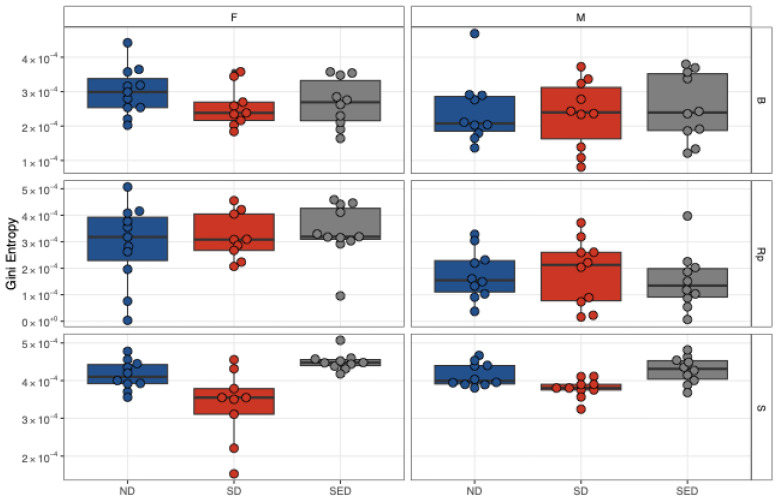
Tissue- and Sex-Specific Genome Diversity of ZIKV Across Dietary Groups. GINI entropy plots depict viral genome diversity in brain (B), reproductive tissue (Rp), and serum (S) samples from female (F) and male (M) mice fed with normal diet (ND), selenium-deficient diet (SD), or selenium plus vitamin E double-deficient diet (SED). Each subplot represents a distinct tissue and sex combination, with GINI entropy values on the *y*-axis and diet groups on the *x*-axis. The data illustrate subtle differences in viral diversity across tissues, sexes, and dietary conditions.

To assess the impact of dietary selenium and vitamin E deficiency on ZIKV genome diversity and mutation accumulation, we analyzed viral sequences from a total 180 samples, including 60 serum samples (20 each from ND, SD, and SED) collected at 6 dpi, 60 reproductive tissue samples (20 each from ND, SD, and SED) collected at 14 dpi, and 60 brain tissue samples (20 each from ND, SD, and SED) collected at 14 dpi from male and female mice maintained on the three dietary regimens (Table 1). Genome diversity was quantified using GINI entropy (Figure 4). No statistically significant differences in overall genome diversity were detected between the three diet groups. However, subtle tissue- and sex-specific variations were observed. In serum samples, the SED group showed a marginally higher genome diversity, while the lowest diversity was observed in the SD group. Analysis of reproductive tissues revealed that ovarian samples consistently exhibited slightly higher diversity than testicular samples across all dietary groups.

### 3.5. Mutation Landscape

Detailed analysis of the viral genomes revealed several mutations, some of which differed from previously reported variants. Notably, the V330L mutation in the E gene was enriched from an allele frequency of 63.51% in the stock virus used for mouse infection to 86.5% and 93.22% in the reproductive tissues and brain of SED-fed mice, respectively. Another notable mutation, D67E in the E gene, was detected in the brain of SED-fed mice. Additionally, the V360I mutation in the NS3 gene was identified in brain and serum samples from both male and female mice within the SED-fed group. A summary of all detected mutations is provided in Table 2.

### 3.6. Production of Neutralizing Antibody

**Figure 5:**
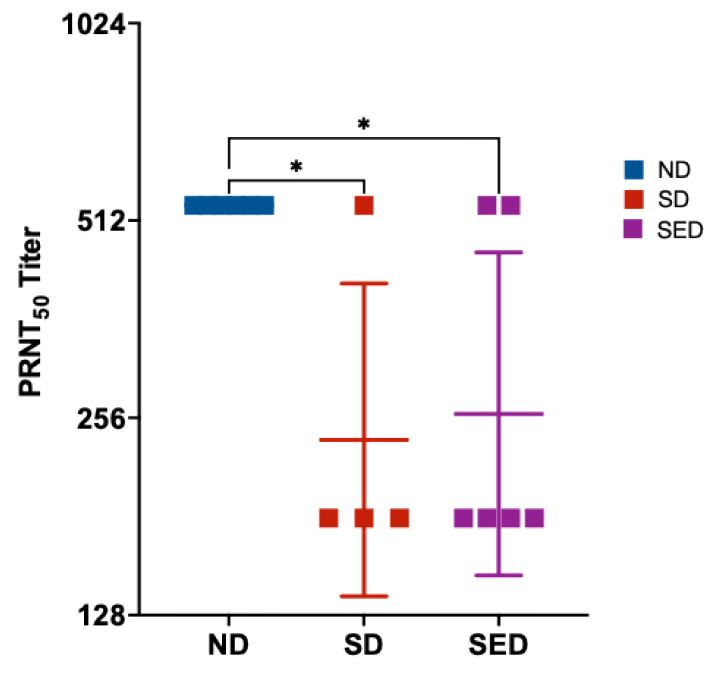
Neutralizing antibody titers (PRNT_50_) in mice from different dietary groups following ZIKV infection. Serum samples were collected 14 days post-infection from mice maintained on a normal diet, selenium-deficient diet, or selenium plus vitamin E double-deficient diet. Neutralizing antibody titers were determined by plaque reduction neutralization test (PRNT). Data are presented as individual values with group means ± SD. * *p* < 0.05 compared to normal diet group.

Selenium and vitamin E are key antioxidants that play a critical role in regulating immune responses, including the production of neutralizing antibodies. Previous studies have demonstrated that deficiencies in these micronutrients impair antibody production and weaken antiviral defenses [5,71,72]. We hypothesized that selenium deficiency, alone or in combination with vitamin E deficiency, would result in reduced neutralizing antibody production in ZIKV-infected mice. Mice maintained on the normal diet exhibited significantly higher neutralizing antibody titers compared to both nutritionally deficient groups. The selenium-deficient and selenium plus vitamin E double-deficient groups showed comparable PRNT_50_ titers, both of which were significantly lower than those observed in the normal diet group (Figure 5). Statistical analysis confirmed that PRNT_50_ titers in the ND group were significantly higher than those in the SD (*p* = 0.0245) and SED groups (*p* = 0.0254).

### 3.7. Cytokine Responses to ZIKV Infection

**Figure 6:**
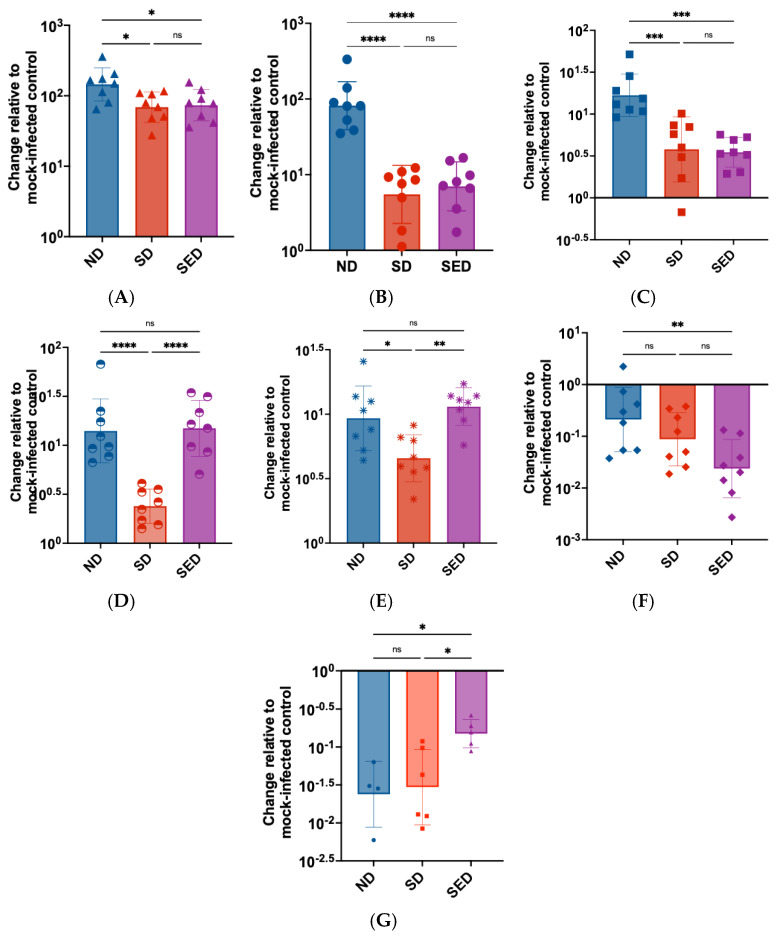
Dietary selenium and vitamin E deficiency modulates antiviral cytokine expression following ZIKV infection. (**A**–**G**) Bar plots of cytokines with significant differences between diet groups: (**A**) IFN-α (**B**) IFN-γ (**C**) TNF-α (**D**) IFN-β (**E**) CCL5 (**F**) IL-12p70 (**G**) GM-CSF. ND: Normal diet; SD: Selenium-deficient diet; SED: Selenium and vitamin E double-deficient diet. N = 8 per diet group. ns: not significant; *: *p* < 0.05; **: *p* < 0.01; ***: *p* < 0.001; ****: *p* < 0.0001.

Cytokine activation is crucial for combatting ZIKV infection, and poor nutritional status can negatively impact expression of antiviral cytokines [73]. Results from the LEGENDplex analysis showed that, although Ifnar1^−/−^ mice lack the type I interferon α/β receptor, they still produced measurable levels of type I interferons (IFN α/β) in response to infection (Supplementary Figure S3). In addition, the cytokine profiles demonstrated distinct patterns of immune modulation across the different dietary groups. In SD and SED mice IFN-⍺, IFN-Ɣ, and TNF-⍺ expression were all significantly reduced compared to ND mice (Figure 6A–C). IFN-β expression was significantly reduced in SD mice but was not significantly changed in the SED mice relative to ND mice (Figure 6D). SD mice showed decreased expression of CCL5 compared to ND (Figure 6E), while SED mice showed decreased expression of IL-12 (Figure 6F) and increased expression of GM-CSF (Figure 6G). Interestingly, several cytokines showed differential expression changes between the deficient diet groups while remaining comparable to expression in ND mice. IFN-β and CCL5 expression were significantly reduced in SD mice but not significantly different between ND and SED mice. Expression was unchanged between ND and SED mice but significantly lower in SD mice. GM-CSF expression was significantly higher in SED mice compared to ND and SD mice, with levels unchanged between ND and SD mice. IL-12(p70) showed a unique trend, where ND and SD expression was not significantly different, SD and SED expression was not significantly different, but SED showed significantly decreased IL-12(p70) expression compared to ND. Other cytokines in the panel showed no significant changes between diet groups (Supplementary Figure S4).

## 4. Discussion

This study demonstrates that combined dietary deficiency of selenium and vitamin E significantly exacerbates ZIKV neuroinvasion and disease severity in Ifnar^−/−^ mice, while having minimal impact on systemic viral spread and overall viral genome diversity. These findings highlight the critical role of antioxidant micronutrients in modulating host susceptibility to neurotropic viral infections [74,75] and reveal important tissue- and sex-specific patterns in ZIKV pathogenesis.

It is important to emphasize that our mouse model, the Ifnar1^−/−^ strain lacking the type I interferon α/β receptor, although widely used for studies of ZIKV pathogenesis because of its high susceptibility to infection and robust viral replication, has inherent limitations. The absence of a functional type I interferon α/β receptor results in compromised innate antiviral signaling, known to be essential for early control of viral infections and influencing subsequent adaptive immune responses [76,77]. Hence, the immune responses, viral dissemination patterns, and tissue tropism observed in IFNAR-deficient mice may differ from those in immunocompetent hosts, including humans, where intact type I interferon responses contribute critically to limiting early viral spread and to the quality and magnitude of T and B cell responses [77,78]. Therefore, while this model is highly valuable for identifying factors that influence ZIKV replication and disease severity under conditions of compromised innate antiviral signaling, the absence of IFN-α/β signaling may limit the generalizability of our findings to natural human infections and to other animal models with intact interferon pathways. Thus, caution is necessary when extrapolating these results to broader populations.

Although both SD and SED diets reduced serum GPx activity compared with ND, GPx activity in SED mice was higher (but not significantly) than in SD mice. A similar trend was observed by Hill et al. [79]. While we do not know the exact reason for this observed pattern, we cautiously presume that this difference, among other factors, may reflect compensatory upregulation of GPx and other antioxidant enzymes in response to increased oxidative stress resulting from the absence of vitamin E, as has been reported in combined selenium and vitamin E deficiency models [5,79]. Importantly, both deficient diets produced clear reductions in GPx activity relative to ND, consistent with effective induction of selenium deficiency [80].

### 4.1. Micronutrient Deficiency Increases ZIKV Neurotropism and Disease Severity

Mice maintained on a selenium and vitamin E double-deficient diet (SED) exhibited markedly higher ZIKV RNA levels in the brain, greater weight loss, and increased mortality compared to those on normal or selenium-deficient diets. This supports the hypothesis that antioxidant micronutrients act synergistically to protect against viral neuroinvasion and neuropathology, similar to reports in other studies [5,7,10,81,82]. The impact of combined deficiency, as opposed to single micronutrient deficiency, is consistent with previous reports that the compensatory antioxidant effect of one micronutrient is lost when both are absent [1,26]. The selective increase in brain viral load, rather than systemic dissemination, suggests that both selenium and vitamin E are particularly important for maintaining central nervous system (CNS) antiviral defenses, potentially through mitigation of local oxidative stress [83,84,85].

### 4.2. Impaired Immune Responses in Deficient Mice

Both the SD and SED groups exhibited significantly reduced neutralizing antibody titers, indicating a compromised adaptive immune response. Vitamin E is one of the most abundant micronutrients in immune cells, where it protects polyunsaturated fatty acids in cell membranes from oxidative damage [71]. Deficiency in vitamin E has been shown to impair humoral and adaptive immune responses, resulting in diminished antibody production and weakened immune defenses [71,72]. Similarly, selenium deficiency has been associated with reduced antibody responses to viral infections, primarily through impaired B cell function and decreased antibody synthesis [5,82]. Despite the reduced antibody titer in both the SD and SED groups, only SED mice developed notable increased brain viral loads and mortality. This suggests that impaired humoral immunity alone is unlikely to account for the enhanced neuroinvasion in SED animals. Instead, these findings suggest a compromise in CNS-specific mechanisms due to combined selenium and vitamin E deficiency. Vitamin E is a major lipid-soluble antioxidant in neural tissues and plays a critical role in protecting polyunsaturated fatty acid–rich membranes from peroxidation, preserving blood–brain barrier integrity, and limiting oxidative injury to neurons and glial cells [23,86]. Increased oxidative stress, occasioned by diminished vitamin E and selenium-dependent antioxidant defenses, could compromise barrier function, facilitate viral entry into the CNS, and alter microglial and astrocyte antiviral responses [87,88,89,90]. A plausible hypothesis is that local oxidative damage and dysregulated glial innate immunity may act in concert with systemic immune impairment to drive the disproportionate CNS viral burden and mortality observed in SED mice.

In addition to reduced antibody titers, mice in both SD and SED groups showed significantly decreased serum levels of key cytokines, including IFN-α, IFN-γ, and TNF-α. Type I interferons, such as IFN-α, are crucial for initiating antiviral innate immune responses [78,91,92]. IFN-γ is produced by antigen-presenting cells and adaptive immune cells in response to infection and plays a major role in activating antiviral CD4+ T helper cells [93]. TNF-α is involved in promoting inflammation, inducing apoptosis of infected cells, and facilitating leukocyte adhesion and migration across the endothelium [92]. The expression of these cytokines is typically upregulated during ZIKV infection, and our data demonstrated that SD and SED diet conditions significantly suppressed their expression. This decrease in major regulatory cytokines can have wide-ranging consequences, including increased tissue damage, impaired immune cell recruitment, and diminished activation of antiviral responses. Notably, the marked reduction in IFN-γ expression is consistent with previous findings that selenium deficiency leads to decreased T cell proliferation and lower serum immunoglobulin levels [94], supporting our observation of reduced ZIKV-neutralizing antibodies in these groups.

For the remaining cytokines in the panel (IL-1β, IL-6, IL-10, IL-12(p70), CCL2, CCL5, CXCL1, CXCL10, and GM-CSF), several were differentially expressed between normal diet and deficient diet groups but generally were not impacted by diet or upstream changes in IFN-γ and TNF-⍺ expression. This is curious given that several of these cytokines are regulated by IFN-γ, and TNF-⍺, such as CCL2, CCL5, IL-1β, IL-6, IL-10, and IL-12(p70) [92]. Our data suggest that expression of interleukins and other chemokines may not be directly impacted by diet background, and if their regulatory cytokines are differentially expressed, alternative mechanisms may allow for their typical expression.

It is important to note that the mouse model used in this study lacks the type I interferon α/β receptor, which prevents the downstream canonical type I interferon signaling cascade necessary for effective antiviral protection. However, this genetic modification does not affect the expression of IFN-α and IFN-β themselves (Figure S4), as the genes encoding these cytokines remain intact. Cytokine responses during viral infection are highly dynamic and shaped by multiple, interconnected pathways. Even in the absence of canonical type I interferon signaling, interferon-stimulated gene expression can still be induced in Ifnar1^−/−^ mice through alternative routes, including TNF-α/NF-κB, cGAS–STING, and double-stranded RNA sensing pathways [95]. Innate immune recognition of ZIKV triggers a broad cytokine program, including type I interferons, CXCL10, CCL5, and IL-1β, among others [96,97], while ZIKV infection of innate immune cells such as macrophages and NK cells can antagonize or modulate these responses [96]. In addition, individual cytokines can amplify or suppress the expression of other mediators, resulting in a highly context- and time-dependent network [96,97]. Our measurements were performed at a single time point, which provides only a snapshot of this dynamic process; longitudinal cytokine profiling at multiple stages of infection would be required to fully define how dietary status shapes the temporal evolution of cytokine responses during ZIKV infection. However, the cytokine expression data presented here remain valid and informative, despite the absence of receptor-mediated signaling [98].

### 4.3. Viral Genome Diversity and Mutation Patterns

Previous studies have demonstrated that deficiencies in antioxidant micronutrients, such as selenium, can drive the evolution of increased viral diversity and virulence in several RNA viruses, including influenza virus, coxsackievirus, and HIV [44,46,61,99]. However, in the present study, we did not observe significant differences in ZIKV genome diversity between mice fed adequate and deficient diets. This contrasting finding suggests that the relationship between antioxidant micronutrient deficiency and viral evolution may not be universal across all viruses. It remains unclear whether selenium and vitamin E deficiencies have no impact on ZIKV evolution in this study, or whether the single infection time point and absence of serial inter-host passages limited the opportunity for the emergence and amplification of the expected viral diversity. Previous research has shown that repeated serial passage can be a critical factor in revealing the effects of host nutritional status on viral evolution [46,61]. Supporting this, data from our laboratory indicate that serially passaged SARS-CoV-2 isolates in selenium-deficient BALB/c mice exhibited markedly increased viral diversity compared to those in mice on a nutritionally adequate diet [100].

Despite the lack of statistically significant differences in overall ZIKV diversity between diet groups, we did observe subtle tissue and sex-specific patterns, with slightly elevated viral diversity in the sera and reproductive tissues of selenium-deficient female mice. This observation is noteworthy, as severe outcomes of ZIKV infection, such as neurological impairment and microcephaly, are linked to vertical transmission during pregnancy [101,102]. We hypothesize that viral populations with greater diversity may be better equipped to establish fetal infection and contribute to the development of severe congenital disease [70,103]. However, further research using pregnant, nutrient-deficient mouse models is needed to clarify the relationship between maternal micronutrient status, ZIKV diversity, and the risk of fetal infection.

### 4.4. Sex-Specific Differences in Viral Load

Male mice consistently exhibited higher viral load in blood and reproductive tissues including mice fed SD and SED diets. The detection of high ZIKV load in the testes at day 14 post-infection indicates persistent infection and viral residence within male reproductive tissues. This finding is particularly significant for sexual transmission, as it suggests that infected males could facilitate the spread of novel variants via an alternate transmission route. The public health implications are considerable, especially if direct host-to-host transmission, combined with nutritional deficiency, contributes to the emergence of more virulent viral strains, as has been observed with other viruses such as influenza and Coxsackie virus [104].

The observed differences in our study likely arose from sex-specific hormonal regulation of immune responses among other factors that may influence ZIKV pathogenesis. Sex hormones modulate both innate and adaptive immunity, contributing to differential viral control and tissue tropism between males and females [105,106]. In males, testicular immune privilege and high ZIKV replication in the male reproductive tract may further shape systemic and local antiviral responses [107,108]. Nutritional deficiencies in selenium and vitamin E may have exacerbated these inherent sex biases by differentially impacting oxidative stress and immune regulation [7,99]. Further research is needed to clarify how sex and micronutrient status interact to shape antiviral immunity in ZIKV infection.

### 4.5. Mutation Analysis and Potential Functional Implications

Several notable mutations were identified in the viral genomes from various tissues and dietary groups in our study. Although these mutations did not correlate with distinct or unique phenotypic manifestations in the host within our experiments, the implications of some have been described in the literature. The V330L mutation in the E gene, previously reported as attenuating in Ifnar1^−/−^ mice infected with the PRVABC59 strain of ZIKV [68,109], was present in the stock virus and detected across all experimental groups. Notably, this substitution appeared at higher allele frequencies in brain and reproductive tissue samples from males in the SED and SD groups, suggesting possible enrichment under deficient conditions. Further investigation is required to more clearly associate this observation with nutritional deficiency, and to understand its biological significance particularly in male mice. Furthermore, the D67E mutation on the E gene was detected in the brain of a female mouse from the SED diet group. Notably, mutation at this position (D67N) has been reported to markedly increase neurovirulence in mice [69], suggesting that substitutions at this site may have important functional implications for viral pathogenesis.

The V360I mutation in the NS3 gene, previously described in other ZIKV isolates [67], was detected in the brain and serum of both male and female mice within the SED group. The implication of this mutation is not known and the fact that it is within the helicase region of the NS3 [110] makes it interesting for future studies.

These mutations were observed in a limited number of animals and were not statistically enriched across multiple hosts. Therefore, they could not be interpreted as solid evidence of selection or adaptive evolution. Our hypothesis is that they may reflect stochastic bottlenecks, tissue compartmentalization, or host-specific replication constraints.

### 4.6. Clinical and Public Health Implications

These findings have important implications for populations at risk of micronutrient deficiencies, particularly in regions endemic for ZIKV and other neurotropic pathogens. Ensuring adequate selenium and vitamin E intake may be critical for reducing the risk of severe neurological complications and improving outcomes during viral outbreaks [5,6]. Nutritional interventions could serve as adjuncts to vaccination and antiviral therapies, especially in vulnerable groups [9].

### 4.7. Limitations and Future Directions

This study provides valuable insights into the effects of antioxidant micronutrient deficiencies on ZIKV infection and viral evolution in mice. However, our experimental model focused on individual animals and did not address viral evolution across multiple hosts within a population. As a result, we were unable to assess how rare mutations might be selected for, maintained, or spread through repeated host-to-host transmission. In addition, three mice deaths were recorded during the study, but samples could not be collected from these animals for processing due to existing animal facility protocols. Therefore, we are unable to determine how information from the mice that died could have impacted the equilibrium of genetic diversity and mutation observed in this research. Future research utilizing population-based or serial passage models will be essential to determine whether such mutations can acquire epidemiological significance over time, as has been observed with serial passage of SARS-CoV-2 in our laboratory. While the use of Ifnar^−/−^ mice provides a sensitive model for studying ZIKV pathogenesis, it may not fully recapitulate the immune responses of immunocompetent hosts. Additionally, while we identified several potentially important viral mutations, their functional consequences, particularly regarding viral fitness, pathogenicity, and drug sensitivity, remain largely unexplored. Follow-up studies using reverse genetics and in vivo challenge models will be necessary to clarify the roles of these variants.

## Figures and Tables

**Table 1:** Description of samples analyzed.

Duration of Experiment	Diet Groups	Number of Mice	Samples Analyzed
Day 0–Day 6	ND	20	Serum *^#π^
	SD	20	Serum *^#π^
	SED	20	Serum *^#π^
Day 0–Day 14	ND	20	Serum ^σ^, Brain *^#^, reproductive tissue *^#^
	SD	20	Serum ^σ^, Brain *^#^, reproductive tissue *^#^
	SED	20	Serum ^σ^, Brain *^#^, reproductive tissue *^#^
Total		120	

Analysis performed: * = qPCR, ^#^ = whole genome sequencing, ^π^ = cytokine profiling, ^σ^ = neutralizing antibody evaluation.

**Table 2:** Notable Mutations Detected in ZIKV Genomes from Mice on Different Diets.

Diet	No.	Sex	Tissue	AF (%)	Gene	AA Pos.	Observed Mut	Reported Mut	Reference
SED	1	F	Rp	4.91	C	106	A106T	T106A	Yu et al. [65]
SD	1	F	S	1.20	C	106	A106V	T106A	
SED	1	F	Rp	13.39	Pr	17	N17S	S17N	Yuan et al. [66]
ND	1	F	Rp	1.71	Pr	17	N17S	S17N	
SED	1	M	B	27.65	E	317	I317L	I317V	Aziz et al. [67]
SD	1	F	Rp	2.43	E	317	I317T	I317V	
SED	1	M	B	93.22	E	330	V330L	V330L	Carbaugh et al. [68]
SED	4	M	Rp	69.2–86.5	E	330	V330L		
SD	1	M	Rp	64.82	E	330	V330L		
Input V	1			63.51	E	330	V330L		
SED	1	F	B	1.04	E	367	D67E	D67N	Liu et al. [69]
SD	1	M	S	1.23	E	487	M487V	T487M	Aziz et al. [67]
SED	1	M	B	3.50	NS1	188	V188A	V188A	Liu et al. [69]
SD	1	M	B	2.53	NS1	349	M349T	M349V	Collette et al. [70]
SED	1	M	B	6.82	NS3	360	V360I	V360I	Aziz et al. [67]
SED	1	F	S	1.18	NS3	360	V360I	V360I	
SD	1	F	S	1.07	NS3	584	H584Q	H584Y	Collette et al. [70]

Abbreviations: ND, normal diet; SD, selenium-deficient diet; SED, selenium plus vitamin E double-deficient diet; Input V, input virus; F, female; M, male; S, serum; Rp, reproductive tissue; B, brain; AF, allele frequency.

## Data Availability

The original data presented in the study are openly available in NCBI Sequence Read Archive under BioProject accession number PRJNA1374194.

## Supplementary Materials

The following supporting information can be downloaded at: https://www.mdpi.com/article/10.3390/v18020177/s1, Table S1. Composition of experimental diets. Table S2. Custom-designed sequencing primers (M1000) for multiplex amplification of Zika virus strains PRVABC59 and FSS13025. Table S3. Mappgene parameters used for sequence processing and variant calling. Figure S1. Quantification of Zika virus RNA genome copies in blood and brain tissues. Figure S2. Sequencing Coverage Profiles Obtained with the M1000 Primer Set. Figure S3. Comparison of cytokine expression in ZIKV-infected versus PBS-treated mice across the dietary groups. Figure S4. Dietary selenium and vitamin E deficiency impacts cytokine responses to Zika virus infection.
