# Supplementary material for: Impact of Selenium and Vitamin E Deficiency on Zika Virus Pathogenesis and Immune Response in Mice
Source: Viruses. 2026 Jan 28;18(2):177. doi: 10.3390/v18020177 (PMC12945010; doi:10.3390/v18020177)
Supplement: Supplementary file 1 [file viruses-18-00177-s001.zip › viruses-4074842-supplementary.pdf]

Impact of Selenium and Vitamin E Deficiency on Zika Virus Pathogenesis and Immune Response in Mice

Supplemental information

Supplementary Table S1. Composition of experimental diets.
The diets were commercially sourced from Teklad Custom Diets, Madison, WI.
A. TD.96363 – Selenium Control Diet (Normal Diet, ND)
B. TD.92163 – Selenium Deficient Diet (SD)
C. TD.240607 – Selenium and Vitamin E Deficient Diet (SED)

**A. TD.96363 – Selenium Control Diet**	
**Formula**	**g/Kg**
Torula Yeast	300.0
DL-Methionine	3.0
Sucrose	591.0
Corn Oil	50.0
Mineral Mix, AIN-76 (170915)	35.0
Calcium Carbonate	11.0
Vitamin Mix, Teklad (40060)	10.0
**B. TD.92163 – Selenium Deficient Diet**	
Torula Yeast	300.0
DL-Methionine	3.0
Sucrose	591.0
Corn Oil	50.0
Mineral Mix, Se Deficient (80313)	35.0
Calcium Carbonate	11.0
Vitamin Mix, Teklad (40060)	10.0
**C. TD.240607 – Selenium and Vitamin E Deficient Diet**	
Torula Yeast	300.0
DL-Methionine	3.0
Sucrose	566.7998
Flaxseed Oil	50.0
Cellulose	30.0
Mineral Mix, Se Deficient (80313)	35.0
Calcium Carbonate	11.0
Choline Dihydrogen Citrate	3.5
Vitamin A Palmitate (500,000 IU/g)	0.06
Vitamin D_3, cholecalciferol (500,000 IU/g)	0.0066
Vitamin B_{12}, (0.1% mannitol)	0.075
Biotin	0.0006
Calcium Pantothenate	0.066
Folic Acid	0.003
Inositol	0.165
Vitamin K3, menadione	0.075
Niacin	0.15
Pyridoxine HCl	0.033
Riboflavin	0.033
Thiamin (81%)	0.033

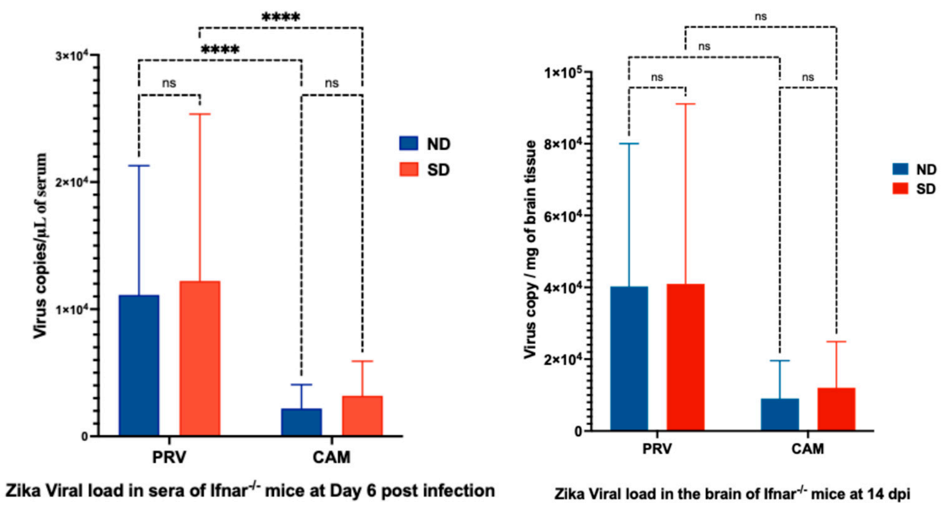

Figure S1.
Quantification of Zika virus RNA genome copies in blood and brain tissues.
(A) Zika virus genome copies in blood at 6 days post-infection.
(B) Zika virus genome copies in brain tissue at 14 days post-infection.
ND, normal diet; SD, selenium-deficient diet; SED, selenium and vitamin E double-deficient diet. Statistical analysis was performed using two-way ANOVA in GraphPad Prism v10.

Supplementary Table S2. Custom-designed sequencing primers (M1000) for multiplex amplification of Zika virus strains PRVABC59 and FSS13025.

Primers were designed based on reference sequence KU501215.1 using the Primal Scheme web-based tool (https://primalscheme.com/).

Oligos start	Oligos end	Zika virus Primer	Primer pool		Sequence (5'-3')	*Amplicon size (bp)*	Overlap size (bp)
10	32	ZikaPRV.FSS_1_LEFT	1	+	TGTGTGAATCAGACTGCGACAG		
1026	1048	ZikaPRV.FSS_1_RIGHT	1	-	GACAACATCAACCCAAGTCCCA	*1038*	
899	921	ZikaPRV.FSS_2_LEFT	2	+	CTTGGCTTTTGGGAAGCTCAAC		149
1872	1894	ZikaPRV.FSS_2_RIGHT	2	-	GGAGTATGACACGCCCTTCAAT	*995*	
1755	1777	ZikaPRV.FSS_3_LEFT	1	+	TCAAGAAGGAGCAGTTCACACG		139
2731	2753	ZikaPRV.FSS_3_RIGHT	1	-	CGACCGTCAGTTGAACTCCATT	*998*	
2607	2629	ZikaPRV.FSS_4_LEFT	2	+	TAGATTGGCAGCAGCAGTCAAG		146
3577	3599	ZikaPRV.FSS_4_RIGHT	2	-	TGAGCAGAATCACAAGCACTCC	*992*	
3485	3507	ZikaPRV.FSS_5_LEFT	1	+	TGGAGATAAGGCCCAGGAAAGA		114
4446	4468	ZikaPRV.FSS_5_RIGHT	1	-	ACCACTCTCATCTAG**Y**GCCACA	*983*	
4338	4360	ZikaPRV.FSS_6_LEFT	2	+	TGTCAGTTACGTGGTCTCAGGA		130
5322	5344	ZikaPRV.FSS_6_RIGHT	2	-	CATATAACGCACTGGAAGCCCT	*1006*	
5227	5249	ZikaPRV.FSS_7_LEFT	1	+	CCTGAAATAGTCCGTGAAGCCA		117
6174	6196	ZikaPRV.FSS_7_RIGHT	1	-	TTGCTCCGTCCTAAGCTTGAAC	*969*	
6063	6083	ZikaPRV.FSS_8_LEFT	2	+	TGCACACTGGCTTGAAGCAA		133
7053	7075	ZikaPRV.FSS_8_RIGHT	2	-	GACGGCTGGGGTAATGAAAGTT	*1012*	
6918	6940	ZikaPRV.FSS_9_LEFT	1	+	ACTCGGATGGTTGGAGAGAACA		157
7940	7962	ZikaPRV.FSS_9_RIGHT	1	-	TCTTGAACTTTGCGGATGGTGG	*1044*	
7844	7866	ZikaPRV.FSS_10_LEFT	2	+	CAAAGCTGAGATGGTTGGTGGA		118
8794	8816	ZikaPRV.FSS_10_RIGHT	2	-	TCCACAACCAGGAAGAGACCAT	*972*	
8674	8696	ZikaPRV.FSS_11_LEFT	1	+	ACTGGAGTCACAGGAATAGCCA		142
9647	9669	ZikaPRV.FSS_11_RIGHT	1	-	ACGCAATCATCTCCACTGACTG	*995*	
9517	9539	ZikaPRV.FSS_12_LEFT	2	+	GTGGTGCA**R**CTCATTCGGAATA		152
10460	10484	ZikaPRV.FSS_12_RIGHT	2	-	CTGACTAT**R**GGCTTGGTTTCCCAG	*967*	

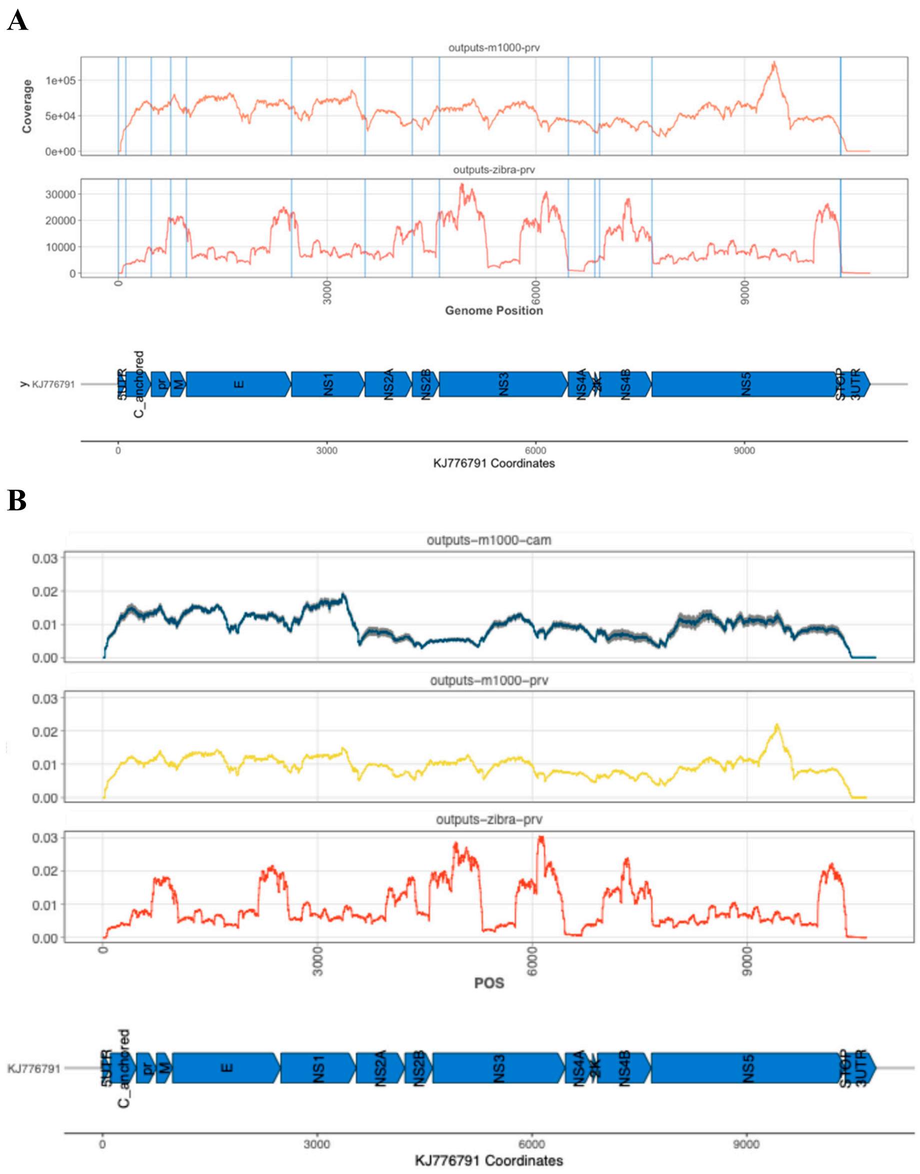

Figure S2. Sequencing Coverage Profiles Obtained with the M1000 Primer Set Panel A shows the raw per-base coverage plots. Panel B shows normalized relative coverage, expressed as percentage of total reads that mapped to each genome position. The M1000 primer set was utilized in amplicon-based whole-genome sequencing of Zika virus samples from the Cambodian strain (output_m1000_cam) and the PRV strain (output_m1000_prv). Sequencing coverage obtained with the M1000 primer was compared with that generated using the previously published ZIBRA primer scheme (output_zibra_prv; reference: doi:10.1186/s13059-018-1618-7), which was originally validated for sequencing of the PRV strain only. The M1000 primer demonstrated improved sequencing coverage across both Zika virus strains.

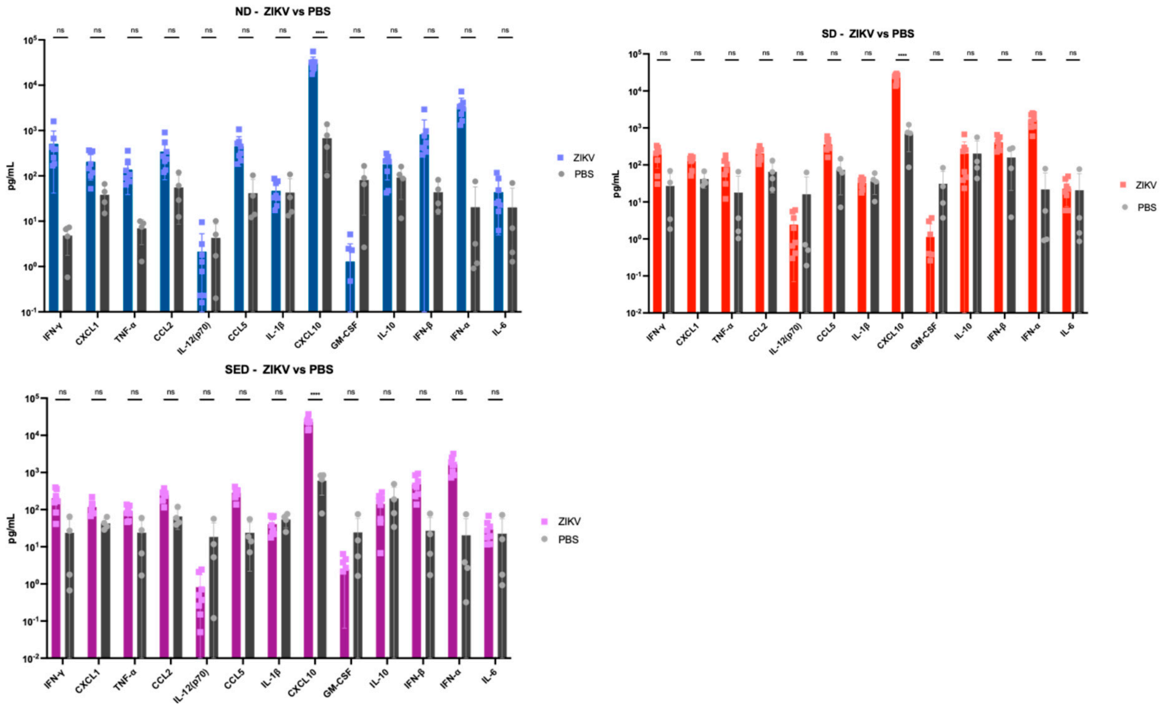

Figure S3. Comparison of cytokine expression in ZIKV-infected (n=8) versus PBS-treated (n=4) mice across the dietary groups

ND: Normal diet; SD: Selenium-deficient diet; SED: Selenium and vitamin E double-deficient diet. Cytokine concentrations were measured using a Legendplex flow cytometric assay. Statistical analysis was performed using two-way ANOVA followed by Šídák's multiple comparisons test. ns,not significant; ****, $p<0.0001$

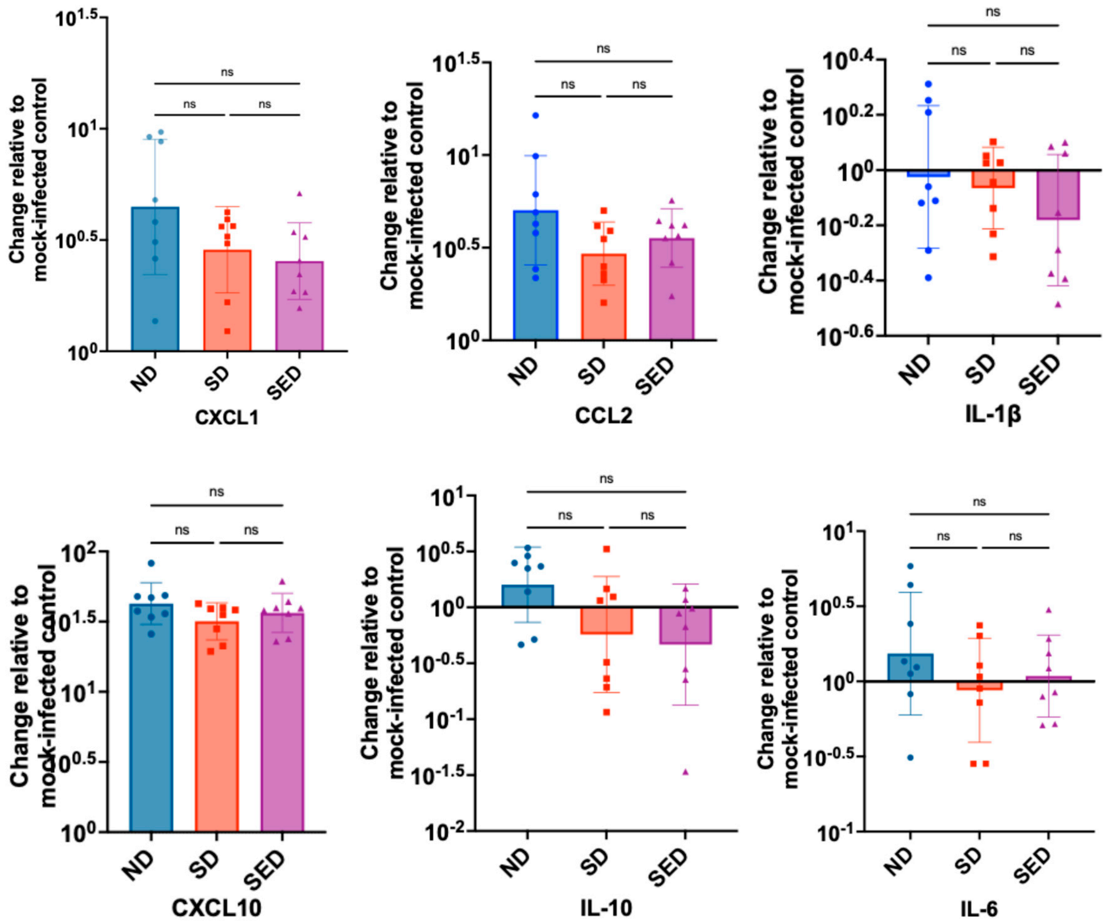

Figure S4. Dietary selenium and vitamin E deficiency impacts cytokine responses to Zika virus infection.

ND: Normal diet; SD: Selenium-deficient diet; SED: Selenium and vitamin E double-deficient diet.

Cytokine concentrations were measured using a Legendplex flow cytometric assay. Statistical analysis was performed using one-way ANOVA followed by Tukey's multiple comparisons test. ns, not significant;

Supplementary Table S3. Mappgene parameters used for sequence processing and variant calling.

Parameter / Option	Description
`--depth_cap 300000`	Consider only 30,000 reads per site in the LoFreq step
`--no-fixq`	Do not adjust base qualities
`--no_ncov --reference_accession KU501215.1 --gff KU501215.1.gff3`	Do not use SARS-CoV-2; use Zika PRVABC59 genome and annotation
`--primers_bp m1000_KU501215.1`	Use M1000 primer regions on PRVABC59 genome
`--trim_front_tail 0`	Skip mappgene's fastp trimming
`--use_full_node`	Use one cluster node per sample
